# Morphologic Characterization of Strongylida Larvae from Human and Swine Coprocultures in Rural Communities in the State of Piauí, Northeastern Brazil

**DOI:** 10.1155/2022/7251922

**Published:** 2022-08-23

**Authors:** Polyanna A. A. Bacelar, Kerla J. L. Monteiro, Jéssica P. dos Santos, Denilson de A. e Silva, Daniella N. Leal, Mayron M. Almeida, Brenda B. C. Evangelista, Francisco M. de Oliveira-Neto, Filipe A. Carvalho-Costa

**Affiliations:** ^1^Laboratory of Epidemiology and Molecular Systematics, Oswaldo Cruz Institute, Oswaldo Cruz Foundation, Rio de Janeiro, Brazil; ^2^Regional Office Oswaldo Cruz Foundation—Piauí, Teresina, Brazil

## Abstract

Some helminth species belonging to the order Strongylida are parasites of the digestive tract of vertebrates, including man and domestic animals. In humans, infections with *Oesophagostomum* spp. and *Trichostrongylus* spp. may be misdiagnosed as hookworm disease on parasitological stool examination, mainly in regions where these infections are coendemic, since eggs released in hosts' feces are morphologically similar. This study presents the morphologic characterization of Strongylida larvae recovered from humans and pigs living in close proximity, exploring putative zoonotic cycles. One hundred three humans and 27 pigs were included in low-resource rural communities in the state of Piauí, northeastern Brazil. Strongylida eggs were present in 12 (11.7%) humans and 23 (85.2%) pigs through conventional parasitological examination. Strongylida-positive fecal samples were submitted to coprocultures using the Harada–Mori technique. All 22 larvae obtained from human feces were classified as hookworms (Ancylostomatidae). From a total of 37 larvae obtained from swine, 23 (62.3%) were classified as *Oesophagostomum*, 6 (16.2%) were *Hyostrongylus*, and 4 (10.8%) were *Trichostrongylus*. Four larvae (10.8%) obtained from pigs were classified as *Strongyloides*. The morphological study of filariform larvae obtained in coprocultures is a useful and inexpensive tool in the screening of intestinal helminthiasis in a One Health approach.

## 1. Introduction

Some helminth species belonging to the order Strongylida (Phylum, Nematoda; Class, Chromadorea) are parasites of the digestive tract of vertebrates, including man and domestic animals [1]. The eggs released in hosts' feces are morphologically similar, with some differences in size, so it is difficult to identify the genus/species by conventional parasitological examination [2].

Strongylida eggs are thin-shelled and designed to release into the environment; after about 24 hours, Rhabditoid larvae survive and grow by actively feeding on the organic matter in the soil. These first-stage larvae, approximately 250 *μ*m in length, undergo a cuticle molt 48 hours after hatching and grow until reaching near 400 *μ*m, giving rise to the second-stage larvae. These continue to grow until reaching 500–700 *μ*m, develop a new sheath, and molt to the third stage (filariform larvae), when they no longer grow or feed, being ready to penetrate the host, percutaneously or orally [3]. Most parasitic Strongylida species are directly transmitted and filariform larvae in the environment represent the infective stage. Within the vertebrate host, and after a new molt, fourth-stage larvae emerge migrating through different organs; most Strongylida species will inhabit the digestive tract of the vertebrate host, where the adult worms copulate and females lay eggs which are shed with the host's feces [3].

The order Strongylida has four families encompassing parasites that infect humans: (i) Ancylostomatidae, which includes hookworms such as *Necator americanus* and *Ancylostoma* spp., (ii) Chabertidae, which includes *Oesophagostomum* spp. and *Ternidens deminutus*, (iii) Trichostrongylidae, to which *Trichostrongylus* spp. belong (and the enzootic species *Hyostrongylus rubidus*, the red stomach worm of pigs, *Haemonchus contortus*, parasite of small ruminants, and the parasites of bovines *Cooperia punctata* and *Ostertagia ostertagi*), and (iv) Angiostrongylidae, which includes *Angiostrongylus cantonensis* and *Angiostrongylus costaricensis*. Besides these families, Metastrongylidae includes *Metastrongylus* spp., the lungworm of pigs, only accidentally transmitted to humans, and the family Syngamidae includes *Syngamus trachea*, which infects birds, and *Mammomonogamus laryngeus*, a parasite of cats, ungulates, and orangutans, which can also rarely infect humans.

Some Strongylida parasites are zoonotic. *Oesophagostomum bifurcum* infects nonhuman primates and humans, being endemic in populations living in some regions of Togo and Ghana, being efficiently mitigated by collective chemoprophylaxis with albendazole [4, 5]. Human infections with *Trichostrongylus* spp. have been reported in populations living in contact with goats and sheep or their feces in many regions [6, 7]. In humans, infections with *Oesophagostomum* spp. and *Trichostrongylus* spp. may be misdiagnosed as hookworm disease on parasitological stool examination [8, 9], mainly in regions where these infections are coendemic.

Although the hookworms which infect humans (*N. americanus* and *Ancylostoma duodenale*) are markedly anthroponotic, cross-host transmission of *N. americanus* among humans and nonhuman primates has been documented in Africa [10, 11]. There is scientific evidence that a potential zoonotic cycle of hookworm disease in the human-swine interface can be considered feasible. In El Salvador, living in close proximity to pigs was a recognized risk factor for hookworm infection [12]. In Ghana, viable *N. americanus* eggs were recovered from pigs' feces [13]. *Necator suillus* is a hookworm species described in pigs for almost a century [14], and investigations into its validity, occurrence, epidemiology, and zoonotic potential have not been forwarded.

The present work is part of a broader project, in which we assessed, through molecular tools (mitochondrial DNA sequencing), the zoonotic circulation of Strongylida and *Ascaris* in the human-swine interface in low-resource rural communities in the state of Piauí [15–18]. In this report, we present the results of the morphological study of Strongylida larvae obtained through cultivation of human and swine feces.

## 2. Materials and Methods

### 2.1. Setting and Study Design

**Figure 1:**
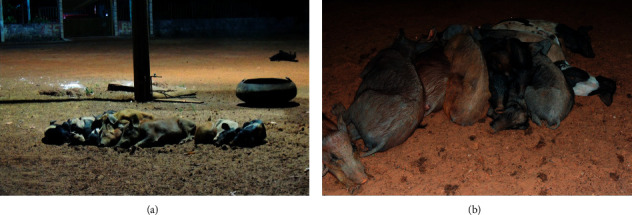
Pigs circulating freely on the streets in the municipality of Nossa Senhora de Nazaré, Piauí, Brazil.

The fieldwork was carried out in rural communities in Nossa Senhora de Nazaré, in the state of Piauí, northeastern Brazil. In the communities studied, pigs are raised extensively and roam freely on the streets, providing soil contamination with fecal matter (Figures 1(a) and 1)(b). The human population practices open defecation and sanitation conditions are poor. Households were visited by the research team to obtain sociodemographic data and human fecal samples in plastic bottles (*n* = 103 residents) without preservatives. Swine fecal samples (*n* = 27 pigs) were obtained after spontaneous defecation (*n* = 16) or necropsy (*n* = 11).

### 2.2. Parasitological Procedures

All fecal samples were screened for Strongylida eggs by conventional parasitological examination, including the Ritchie, Kato-Katz, and flotation in hypertonic glucose solution techniques [19–22]. Strongylida-positive fecal samples were submitted to coprocultures using the Harada–Mori technique [23]. Briefly, 1-2 g of feces was spread on a 150 × 15 mm filter paper. This strip was placed in a 20 × 200 mm Falcon tube containing 2-3 mL of distilled water. The tube was closed and kept in an upright position for approximately 10 days. Finally, the water was examined with a magnifying glass to check for the presence of larvae. Larvae were collected with Pasteur pipettes. The larvae were examined by light microscopy, and the morphological characteristics proposed in the taxonomic key presented in the chart were verified. Larvae were measured with ImageJ (U. S. National Institutes of Health, Bethesda, Maryland, USA). The ANOVA of the length of the larvae, with and without sheath, was performed and plotted in a graph using the software SPSS Statistics for Windows, version 26 (SPSS Inc., Chicago, Ill., USA).

## 3. Results

**Figure 2:**
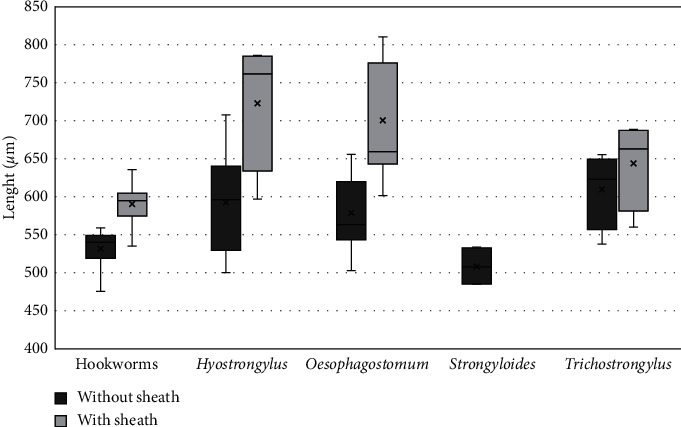
Box plots illustrating the length of filariform larvae of different parasites recovered from human and swine coprocultures by the Harada–Mori technique, with and without the sheath. Comparing the length averaged by ANOVA, the following results are obtained: in the sheath, hookworms vs. *Trichostrongylus*, *p*=0.166; hookworms vs. *Oesophagostomum*, *p* < 0.001. Without the sheath, hookworms vs. *Trichostrongylus*, *p*=0.051; hookworms vs. *Oesophagostomum*, *p* < 0.001. X on plot = mean; transverse line on plot = median.

From a total of 103 residents who provided fecal samples, 12 (11.7%) were positive for Strongylida eggs, and 36 larvae were recovered, of which 22 could be submitted to morphological analysis. Regarding the 27 pigs studied, 16 had their feces collected after spontaneous defecation, and 11 had their feces collected at postslaughter necropsy. Of the total, 23 (85.2%) were positive for Strongylida eggs, and 87 larvae were recovered, of which 37 could be morphologically analyzed. All 22 larvae obtained from human feces were classified as hookworms (Ancylostomatidae). From a total of 37 larvae obtained from swine feces by coproculture, 23 (62.3%) were classified as belonging to the genus *Oesophagostomum*, 6 (16.2%) were *Hyostrongylus*, and 4 (10.8%) were *Trichostrongylus*. Four larvae (10.8%) from pigs were classified as *Strongyloides*. The lengths of the analyzed larvae (mean and standard deviation) are shown in Figure 2.

**Figure 3:**
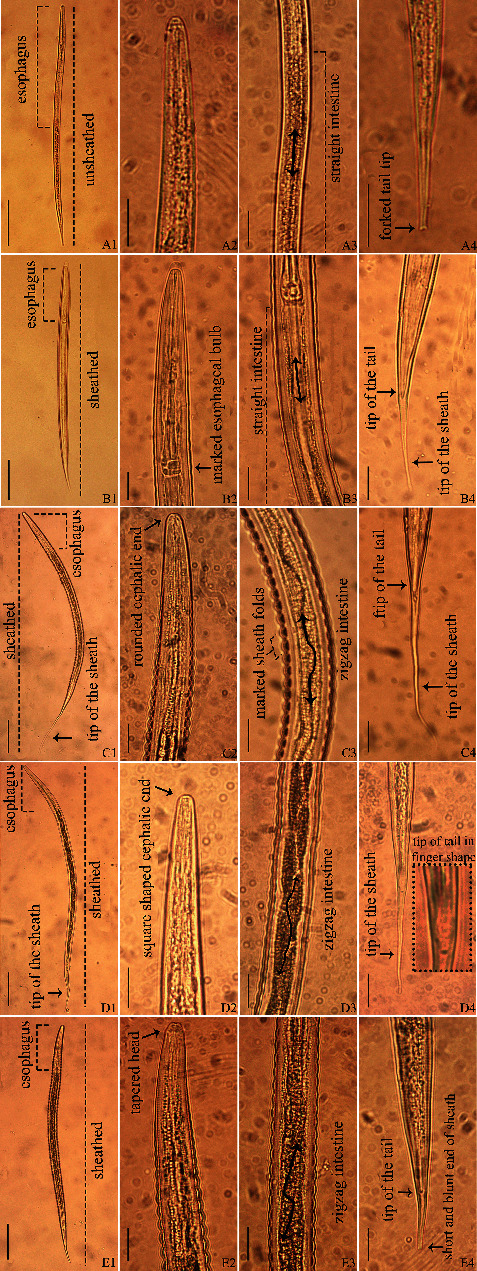
(a1)–(a4) *Strongyloides* larva obtained from swine. (a1). Long esophagus (approximately half the length of the larva), absence of sheath; (a3) straight intestine; (a4) forked tail end. (b1)–(b4) Hookworm larva obtained from human. (b1) Short esophagus (reaching 1/3 of the length of the larva), presence of sheath; (b2) marked esophageal bulb; (b3) straight intestine; (b4) tip of the tail. (c1)–(c4) *Oesophagostomum* larva obtained from swine. (c1) Short esophagus and presence of sheath; (c2) rounded cephalic end; (c3) accentuated sheath folds, zigzag intestine; (c4) long sheath. (d1)–(d4) *Hyostrongylus* larva obtained from swine. (d1) Short esophagus, presence of sheath; (d2) cephalic end in square shape; (d3) zigzag intestine; (d4) tip of the tail in finger shape. (e1)–(e4) *Trichostrongylus* larva obtained from swine. (e1) Short esophagus and presence of sheath; (e2) tapered cephalic end; (e3) zigzag intestine; (e4) short and blunt end of the sheath. A vertical bar in a1, b1, c1, d1, and e1 = 100 *μ*m. Bar in the other images = 20 *μ*m.

*Oesophagostomum* larvae recovered from swine were significantly longer than hookworm larvae obtained from human feces. *Trichostrongylus* larvae also tended to be longer than hookworm larvae. The morphological characteristics of the larvae are shown in Figure 3. An identification key was proposed, based on morphological characteristics for the filariform larvae of some geohelminths of the order Strongylida in humans and swine (Supplementary Table 1).

## 4. Discussion

The present work demonstrates the feasibility of evaluating, through an accessible and inexpensive technique, the genus of Strongylida parasites infecting humans and domestic animals in scenarios where there could be zoonotic transmission and potential misidentification of eggs in fecal microscopic examinations.

No larvae of parasites usually identified in animals were detected among humans, that is, all larvae recovered from humans were morphologically characterized as hookworms. According to Hayashi et al. [24], the length of filariform (third stage) larvae can distinguish *N. americanus* (590–660 *μ*m) from *Ancylostoma duodenalis* (660–720 *μ*m). The length of all hookworm larvae analyzed was compatible with *N. americanus*.

In previous works, we demonstrated that human hookworm disease is endemic in the studied area and that the species involved in the vast majority of cases was *N. americanus*, through DNA sequencing of the parasite's cytochrome oxidase 1 coding gene (*cox*1) fragment [15, 25]. Nevertheless, in some hookworm-positive fecal samples, it was not possible to characterize the *Necator* species present in the material [15]. Previous reports on swine hookworms also motivated the investigation of the presence of *Necator* in pigs in the region [14, 26–29]. The results of the studies on pigs' hookworms carried out in the 1920s and 1930s are conflicting, and it has not been clearly defined whether the hookworm species *N. suillus* is valid or whether those infections were caused by *N. americanus*.

In the present study, the Strongylida genera infecting pigs were *Oesophagostomum*, *Hyostrongylus*, and *Trichostrongylus*, parasites usually present in swine extensively raised. In a previous work, we demonstrated that the *Oesophagostomum* species characterized in pigs in the region by *cox*1 partial sequencing were *O. dentatum*, *O. quadrispinulatum*, and *O. columbianum*, although the latter is a parasite usually found in goats and sheep [17].

*Hyostrongylus rubidus*, the red stomach worm, causes gastritis in pigs and is endemic in several regions, and its occurrence in swine has been described in Brazil [30]. Usually, its presence in herds is verified by the observation of gastric adult worms obtained after slaughter or suspected by the finding of Strongylida eggs in parasitological examinations of feces. The present report illustrates the feasibility of identifying *Hyostrongylus* larvae in swine Harada–Mori coprocultures.

Species belonging to the genus *Trichostrongylus*, mainly *T. colubriformis* and *T. axei* are usually found in small ruminants (goats and sheep) and also parasites and the digestive tract of pigs in many regions [31, 32]. In the studied locality, the close proximity in which small ruminants and pigs are raised may favor cross-host transmission of *Trichostrongylus*, which was detected infecting pigs in the present study. About 10% of the specimens studied had characteristics of *Strongyloides* filariform larvae. Swine strongyloidiasis, caused by *Strongyloides ransomi*, is a worldwide distributed disease more frequent in herds with extensive breeding, determining economic losses for breeders [33].

## 5. Conclusions

The morphological study of larvae obtained by coprocultures is a useful tool in the screening of intestinal helminthiasis and can help the characterization of zoonotic transmission cycles in a One Health approach in socioenvironmental scenarios of poor sanitation and close coexistence of humans and farm animals.

## Data Availability

The data used to support the findings of this study are available from the corresponding author upon request.

## Supplementary Materials

Supplementary Materials Supplemental Table 1: Identification key based on morphological characters for the filarioid larvae of some geohelminths of the order Strongylida in humans and swine [3, 34].

## References

[1] Durette-Desset M. C., Beveridge I., Spratt D. M. (1994). The origins and evolutionary expansion of the Strongylida (Nematoda). *International Journal for Parasitology*.

[2] Ziem J. B., Olsen A., Magnussen P. (2006). Distribution and clustering of *Oesophagostomum bifurcum* and hookworm infections in northern Ghana. *Parasitology*.

[3] Pessoa S. B., Vianna M. A. (1982). *Parasitologia Médica, Guanabara Koogan*.

[4] Ziem J. B., Spannbrucker N., Magnussen P. (2005). *Oesophagostomum bifurcum*-induced nodular pathology in a highly endemic area of Northern Ghana. *Transactions of the Royal Society of Tropical Medicine and Hygiene*.

[5] Polderman A. M., Eberhard M., Baeta S., Horton J. (2010). Chapter 3—the rise and fall of human oesophagostomiasis. *Advances in Parasitology*.

[6] Ghanbarzadeh L., Saraei M., Kia E. B., Amini F., Sharifdini M. (2019). Clinical and haematological characteristics of human trichostrongyliasis. *Journal of Helminthology*.

[7] Lattes S., Ferte H., Delaunay P. (2011). *Trichostrongylus colubriformis* nematode infections in humans, France. *Emerging Infectious Diseases*.

[8] Yong T. S., Lee J. H., Sim S. (2007). Differential diagnosis of *Trichostrongylus* and hookworm eggs via PCR using ITS-1 sequence. *Korean Journal of Parasitology*.

[9] Pit D. S. S., De Graaf W., Snoek H., De Vlas S. J., Baeta S. M., Polderman A. M. (1999). Diagnosis of *Oesophagostomum bifurcum* and hookworm infection in humans: day-to-day and within-specimen variation of larval counts. *Parasitology*.

[10] Kalousová B., Hasegawa H., Petrželková K. J., Sakamaki T., Kooriyma T., Modrý D. (2016). Adult hookworms (*Necator* spp.) collected from researchers working with wild western lowland gorillas. *Parasites and Vectors*.

[11] Hasegawa H., Shigyo M., Yanai Y. (2017). Molecular features of hookworm larvae (*Necator* spp.) raised by coproculture from Ugandan chimpanzees and Gabonese gorillas and humans. *Parasitology International*.

[12] Corrales L. F., Izurieta R., Moe C. L. (2006). Association between intestinal parasitic infections and type of sanitation system in rural El Salvador. *Tropical Medicine and International Health*.

[13] Boyko R. H., Marie Harrison L., Humphries D. (2020). Dogs and pigs are transport hosts of *Necator americanus*: molecular evidence for a zoonotic mechanism of human hookworm transmission in Ghana. *Zoonoses and Public Health*.

[14] Ackert J. E., Payne F. K. (1923). Investigations on the control of hookworm disease. xii. studies on the occurrence, distribution and morphology of *Necator suillus*, including descriptions of the other species of *Necator*. *American Journal of Epidemiology*.

[15] Monteiro K. J. L., Jaeger L. H., Nunes B. C. (2019a). Mitochondrial DNA reveals species composition and phylogenetic relationships of hookworms in northeastern Brazil. *Infection, Genetics and Evolution*.

[16] Monteiro K. J. L., Calegar D. A., Santos J. P. (2019). Genetic diversity of *Ascaris* spp. infecting humans and pigs in distinct Brazilian regions, as revealed by mitochondrial DNA. *PLoS One*.

[17] Bacelar P. A. A., Monteiro K. J. L., Calegar D. A. (2022). Cytochrome c oxidase subunit 1 gene reveals species composition and phylogenetic relationships of *Oesophagostomum* spp. infecting pigs in northeastern Brazil. *Brazilian Journal of Veterinary Parasitology*.

[18] Bacelar P. A. A., Jaeger L. H., Calegar D. A. (2022). Molecular detection of *Metastrongylus salmi* eggs from pigs in low-resource communities in the state of Piauí, northeastern Brazil. *Journal of Veterinary Diagnostic Investigation*.

[19] Young K. H., Bullock S. L., Melvin D. M., Spruill C. L. (1979). Ethyl acetate as a substitute for diethyl ether in the formalin-ether sedimentation technique. *Journal of Clinical Microbiology*.

[20] Katz N., Chaves A., Pellegrino J. (1972). A simple device for quantitative stool thick-smear technique in *Schistosomiasis mansoni*. *Journal of the Institute of Tropical Medicine of São Paulo*.

[21] Sheather A. L. (1923). The detection of intestinal protozoa and mange parasites by a floatation technique. *Journal of Comparative Pathology and Therapeutics*.

[22] Willis H. H. (1921). A simple levitation method for the detection of hookworm ova. *Medical Journal of Australia*.

[23] Harada U., Mori O. A. (1955). A new method for culturing hookworm. *Yonago Acta Medica*.

[24] Hayashi S., Tanaka H., Shirasaka R. (1958). Application of test-tube cultivation method on the survey of bookworm and related human nematodes infection. *Japanese Journal of Experimental Medicine*.

[25] Monteiro K. J. L., Reis E. R. C. D., Nunes B. C. (2018). Focal persistence of soil-transmitted helminthiases in impoverished areas in the State of Piaui, Northeastern Brazil. *Journal of the Institute of Tropical Medicine of São Paulo*.

[26] Buckley J. J. C. (1933). *Necator suillus* as a human infection. *British Medical Journal*.

[27] Buckley J. J. C. (1935). Some observations on Necator suillus Ackert and Payne 1922. *Journal of Helminthology*.

[28] Gordon R. M. (1922). The occurrence of Ankylostomes resembling Necator americanus amongst domestic pigs in Amazonas. *Annals of Tropical Medicine and Parasitology*.

[29] Goodey T. (1923). *Necator americanus* and the domestic pig. *Journal of Helminthology*.

[30] Mendonça R. P. D., Carneiro D. O., Baccin E. M. (2022). Anthelmintic efficacy of oxibendazole against gastrointestinal nematodes in swine. *Brazilian Journal of Veterinary Parasitology*.

[31] Nganga C. J., Karanja D. N., Mutune M. N. (2008). The prevalence of gastrointestinal helminth infections in pigs in Kenya. *Tropical Animal Health and Production*.

[32] Maganga G. D., Kombila L. B., Boundenga L. (2019). Diversity and prevalence of gastrointestinal parasites in farmed pigs in Southeast Gabon, Central Africa.

[33] Roesel K., Dohoo I., Baumann M., Dione M., Grace D., Clausen P. H. (2017). Prevalence and risk factors for gastrointestinal parasites in small-scale pig enterprises in Central and Eastern Uganda. *Parasitology Research*.

[34] Rey L., Parasitology (2013). *Guanabara Koogan*.

