# Supplementary material for: Morphologic Characterization of Strongylida Larvae from Human and Swine Coprocultures in Rural Communities in the State of Piauí, Northeastern Brazil
Source: J Trop Med. 2022 Aug 23;2022:7251922. doi: 10.1155/2022/7251922 (PMC9427283; doi:10.1155/2022/7251922)
Supplement: Supplementary Materials — Supplemental Table 1: Identification key based on morphological characters for the filarioid larvae of some geohelminths of the order Strongylida in humans and swine [3, 34]. [file 7251922.f1.docx]

| **No.** | **Characteristics** | **Identification** |
| --- | --- | --- |
| 1 | Unsheathed body, long esophagus (approximately half the length of the body), straight intestine, forked tail tip | *Strongyloides* spp. |
| 1’ | Sheathed body, medium length esophagus (reaching approximately 1/4 of the body) | 2 |
| 2 | Straight intestinal lumen, marked esophageal bulb, pointed conical tail, tip of the sheath with little filamentous and short, sheath in the anterior region usually away from the head | Hookworm |
| 2’ | Zigzag intestine | 3 |
| 3 | Posterior region of the sheath very filamentous and long, symmetrical folds from the outside of the sheath quite accentuated, tip of the tail conical and with an oval end, rounded head | *Oesophagostomum* spp. |
| 3’ | Short sheath posterior region | 4 |
| 4 | End of the sheath pointed and not very filamentous, square shaped cephalic end, tip of tail in finger shape | *Hyostrongylus* spp. |
| 4’ | Short and blunt end of sheath, tapered head, conical and pointed tail tip | *Trichostrongylus* spp. |

Supplemental Table 1: Identification key based on morphological characters for the filarioid larvae of some geohelminths of the order Strongylida in humans and swine.
